# Bioprocess Engineering Aspects of Sustainable Polyhydroxyalkanoate Production in Cyanobacteria

**DOI:** 10.3390/bioengineering5040111

**Published:** 2018-12-18

**Authors:** Donya Kamravamanesh, Maximilian Lackner, Christoph Herwig

**Affiliations:** 1 Institute of Chemical, Environmental and Bioscience Engineering, Research Area Biochemical Engineering, Technische Universität Wien, 1060 Vienna, Austria; donya.kamravamanesh@tuwien.ac.at; 2 Lackner Ventures and Consulting GmbH, Hofherr Schrantz Gasse 2, 1210 Vienna, Austria; kontakt@drlackner.com; 3 Institute of Industrial Engineering, University of Applied Sciences FH Technikum Wien, Höchstädtplatz 6, 1200 Vienna, Austria

**Keywords:** polyhydroxyalkanoate (PHA), bioprocess design, carbon dioxide, cyanobacteria, upstream processing

## Abstract

Polyhydroxyalkanoates (PHAs) are a group of biopolymers produced in various microorganisms as carbon and energy reserve when the main nutrient, necessary for growth, is limited. PHAs are attractive substitutes for conventional petrochemical plastics, as they possess similar material properties, along with biocompatibility and complete biodegradability. The use of PHAs is restricted, mainly due to the high production costs associated with the carbon source used for bacterial fermentation. Cyanobacteria can accumulate PHAs under photoautotrophic growth conditions using CO_2_ and sunlight. However, the productivity of photoautotrophic PHA production from cyanobacteria is much lower than in the case of heterotrophic bacteria. Great effort has been focused to reduce the cost of PHA production, mainly by the development of optimized strains and more efficient cultivation and recovery processes. Minimization of the PHA production cost can only be achieved by considering the design and a complete analysis of the whole process. With the aim on commercializing PHA, this review will discuss the advances and the challenges associated with the upstream processing of cyanobacterial PHA production, in order to help the design of the most efficient method on the industrial scale.

## 1. Introduction

Petroleum-based polymers are relatively inert, versatile, and durable; therefore, they have been used in industry for more than 70 years [1]. However, they bear negative properties such as CO_2_ emissions from incineration, toxicity from additives, and accumulated toxic substances in the environment, particularly in marine as microplastics, recalcitrance to biodegradation, and massive waste accumulation into the marine environment and the landfills [2,3]. With the limited fossil fuel resources and the environmental impact associated with the products, the research for an alternative seems essential in order to reduce our dependencies on non-renewable resources [4,5].

Biodegradable polymers, due to their eco-friendly nature, offer one of the best solutions to environmental problems caused by synthetic polymers [5]. Polyhydroxyalkanoates (PHAs) are a class of naturally occurring polymers produced by microorganisms [1,6,7], among which poly (3-hydroxybutyrate) (PHB) is the most studied biodegradable polymer that accumulates in bacteria in the form of inclusion bodies as carbon reserve material when cells grow under stress conditions [5,8]. PHB with a high crystallinity represents properties similar to synthetic polyesters and also to polyolefins such as polypropylene [6,9,10]. In addition, due to biocompatibility and biodegradability, PHB possesses extensive interesting functions and can replace fossil-based plastics in many applications [7]. However, the low elongation and break and the brittleness of PHB are limitations that can be overcome using other PHA, like blends of copolymers such as polyhydroxyvalerate (PHV) and poly (3-hydroxybutric acid-co-3-hydroxyvaleric acid) (PHBV). The copolymer can either be directly biosynthesized under varying cultivation conditions or be chemically produced in vitro. Apart from short-chain length PHA, there are medium- and long-chain-length polymers which can help to tailor the material properties [11]. The strategies to overcome these limitations are studied in various wild-type and recombinant cyanobacteria, reviewed by Lackner et al. and Balaji et al. [11,12]. 

Today, PHB is commercially produced by heterotrophic bacteria, such as *Cupriavidus necator* (*C. necator*), and recombinant *Escherichia coli* (*E. coli*) [6,13,14]. High production cost, when compared with petroleum-based polymers, is one major challenge for extensive production and commercialization of PHB [5]. Major contributors to the overall cost being the expensive substrates, continuous oxygen supply, equipment depreciation, high energy demand, and chemicals used for downstream processing [15,16,17].

Attention has been focused to reduce the production cost, mostly by selecting more economically feasible and efficient carbon substrates for PHB production such as whey, hemicellulose, sugar cane, agricultural wastes, and molasses [5,18,19,20]. In this context, PHB production using cyanobacteria from more sustainable resources, such as CO_2_, has gained importance. Cyanobacteria are an ideal platform for the production of biofuels and bulk chemicals through efficient and natural CO_2_ fixation [21].

Other reviews have mainly discussed the potential of cyanobacteria for PHA production, cultivation conditions, and cyanobacterial metabolism, as well as the applications and industrial prospects of the synthesis of this biopolymer [22,23,24,25,26]. Minimization of the PHA production cost can only be achieved by considering the design and a complete analysis of the whole process [27]. In this work, the authors will discuss the bioprocess engineering aspects that focus on upstream processing and advances of sustainable PHA production from cyanobacteria, concentrating primarily on the unit operations of the upstream processing. The authors believe that a proper-time resolved quantification of the process will aid in a better understanding for process manipulation and optimization of industrial production. 

## 2. Cyanobacteria: The Future Host in Biotechnology

Cyanobacteria are gram-negative bacteria with a long evolutionary history and are the only prokaryotes capable of plant-like oxygenic photosynthesis [28]. Unlike heterotrophic organisms, cyanobacteria require only greenhouse gas CO_2_ and sunlight, along with minimal nutrients for growth, eliminating the cost of carbon source and complex media components [28]. Cyanobacteria are equipped with superior photosynthetic machinery, showing higher biomass production rates compared to plants and can convert up to 3–9% of the solar energy into biomass [28,29]. Moreover, in contrast to plants, cultivation of cyanobacteria requires less land area, therefore cyanobacteria do not compete for arable land used for agriculture [30]. Some cyanobacteria can produce PHB, when essential nutrients for growth, such as nitrogen and phosphorus, is limiting. From an economic point of view, however, photosynthetic PHB production in cyanobacteria has two major disadvantages: little productivity and slow growth [25]. Therefore, in order to promote photosynthetic PHB production on an industrially relevant scale, the productivity needs to improve significantly. Productivity is defined as the amount of PHA produced by unit volume in unit time [27]. In spite of all the efforts done, so far, very few reports have shown an actual improvement in the cyanobacterial PHB production process, while there are various challenges associated with cultivation, engineering and large-scale production of autotrophic cyanobacterial biomass.

## 3. Challenges in Cyanobacterial Bioprocess Technology

**Figure 1:**
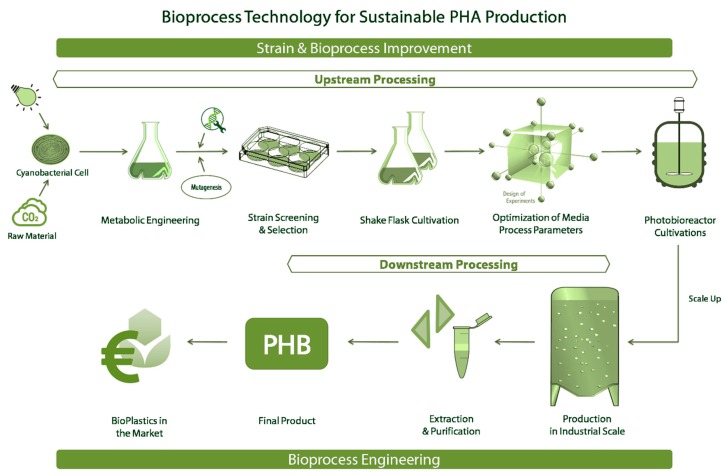
Represents the work-flow in bioprocess technology for the production of polyhydroxyalkanoates (PHAs) using cyanobacteria as the host system.

Figure 1 represents a bioprocess development chain for cyanobacterial PHA production consisting of strain and bioprocess developments and the downstream processing. The workflow shows the strain selection, strain improvement, process understanding, and strategies for scale-up and then down-stream processing, representing the separation and purification of the final product. In order to obtain an optimal scalable bioprocess and in-depth understanding of the bioprocess kinetics, the following points have to be known. First, the holistic knowledge of enzymatic and metabolic pathways of the PHB biosynthesis in cyanobacteria. Second, the selection or development of the optimal strain with maximum productivity. Third, the selection of inexpensive substrate and optimization of media components for the particular production strain. Fourth, the design of the bioreactor system and the optimization of process parameters for scale-up, using the statistical design of experiments (DoE). Fifth, the usage of past data and advanced mathematical models for monitoring and control. Lastly, the development of novel strategies for PHB recovery with a minimum cost of energy and chemical requirement. This review will mainly focus on the bioprocess engineering aspects of photosynthetic PHB production providing an overview of the PHB production process chain starting from a single cell. 

### 3.1. Process Design and Optimization

#### 3.1.1. Existing Wild-Type Strains and Their Reported PHB Content

Cyanobacteria are indigenously the only organisms that produce PHA biopolymers using oxygenic photosynthesis [24]. Cyanobacteria grow mainly under autotrophic conditions, nevertheless, supplementation of sugars or organic acids in some species increases growth and PHB accumulation [24], which contributes to the production cost. To date, a few cyanobacterial strains have been identified for photosynthetic PHB accumulation. Table 1 presents the wild-type cyanobacterial strains, their PHB content in dry cell weight (DCW), and the carbon source used for the production.

As is indicated in Table 1, the PHB production process using CO_2_ as the only carbon source shows a lower product content than using organic acids or sugars as substrate. 

#### 3.1.2. More Competent Cyanobacterial Cell Lines

Cyanobacteria are considered a sustainable and alternative host for PHB production due to their photoautotrophic nature [46]. Despite the fact that cyanobacterial PHB has been the subject of research for many years, it has not found its way to the market. One of the main challenges for cyanobacterial products to enter the market is that cyanobacterial strains are not yet optimized as cell factories for industrial processes. Intensive research has been done over the past 20 years for cyanobacterial strain improvement, research that has aimed to increase PHB productivity, mainly by overexpression of PHB biosynthetic genes. However, these attempts have rarely shown success regarding increased volumetric or specific polymer content for commercial production of cyanobacterial PHB. Recently, Katayama et al. reviewed the production of bioplastic compounds using genetically modified and metabolically engineered cyanobacteria [47]. In this study, we provide a list of genetically modified cyanobacteria with their PHB content and the tools used for the metabolic engineering of the strain. 

#### 3.1.3. Genetic Engineering of Cyanobacteria for PHB Production

Being prokaryotic, cyanobacteria possess a relatively simple genetic background which eases their manipulation [48]. Most cyanobacterial studies on metabolic engineering and PHB biosynthesis have been conducted with a limited number of model strains, of which *Synechocystis* sp. PCC 6803 is the most widely studied species for cyanobacterial research. The research, which has been done for decades on photosynthesis and the genome annotations, has resulted in a wide range of metabolic engineering tools and extensive biological insight for this species [48,49]. PHB production in cyanobacteria occurs mainly via three biosynthetic steps, where two molecules of acetyl-CoA form one molecule of acetoacetyl-CoA using the enzyme 3-ketothiolase encoded with the *phaA* gene [50]. Later, acetoacetyl-CoA is reduced by PhaB to hydroxybutyryl-CoA, utilizing NADPH as an electron donor [51]. In the end, the PHA synthase comprises of PhaC and PhaE polymerizes (R)-3-hydroxybutyryl-CoA to PHB [50,52]. Table 2 summarizes the efforts to overcome the bottlenecks in PHB biosynthetic pathway in cyanobacteria.

Metabolic engineering of cyanobacteria with the aim to increase PHB content was also done by introducing the PHA synthase gene from *C*. *necator* into *Synechhocytis* sp. PCC 6803 [54]. The resulting recombinant *Synechhocytis* sp. PCC 6803 showed increased PHA synthase activity; the total PHB content, however, did not increase [47,54]. For cyanobacterial strain *Synechocystis* sp. PCC 6803, up to 35% (DCW) PHB was obtained using phaAB overexpression and 4 mM acetate [46]. However, the specific production rates in this case also did not show a significant improvement either. Recently, the overexpression of the acetoacetyl-CoA reductase gene in *Synechocystis* was found to increase the productivity of R-3-hydroxybutyrate from CO_2_ to up to 1.84 g L^−1^ [58]. The highest volumetric productivity reported in this case was 263 mg L^−1^ d^−1^.

#### 3.1.4. Randomly Mutated Strains with Improved PHB Content

As an alternative approach to genetic engineering, random mutagenesis can be done for the generation of a mutant library with improved phenotypes. Mutagenesis can be done by exposing the cells of interest to a mutagenic source in order to induce random mutations into the genome. This can, for instance, completely knock-out a gene function [60] or increase enzymatic activity. UV irradiation is the most frequently used mutagen, which leads to transversion in the genome. Furthermore, ethidium bromide and ethyl-methanesulfonate are used as chemical mutagens [60,61]. A major disadvantage of using random mutagenesis is the need for intensive screening to select the mutant with desired phenotypes. The cyanobacterial strain *Synechocystis* sp. PCC 6714 has a great potential as photosynthetic PHB production organism. It has shown up to 17% (DCW) PHB content under nitrogen and phosphorus limiting conditions [32]. In addition to PHB, the strain also accumulates glycogen during the early phase of nitrogen limitation [62]. The random mutagenesis approach used for *Synechocystis* sp. PCC 6714 showed an increase in productivity of up to 2.5-folds resulting in 37 ± 4% (DCW) PHB for the best mutant [63]. The UV-mutation lead to an amino acid change in the phosphate system transport protein (PstA), resulting in higher efficiency of photosynthesis and CO_2_ uptake rate for the mutant MT_a24 [63]. 

#### 3.1.5. CRISPR/Cas Based Genome Editing in Cyanobacteria

Cyanobacteria are promising platforms for the production of biofuels and bio-based chemicals, however, the metabolic engineering of cyanobacteria poses various challenges [64]. CRISPR/Cas technology has enabled genome modification of cyanobacteria with gene substitution, marker-less point mutations, and gene knockouts and knock-ins with improved efficiency [65]. So far, the CRISPRi system has been used to downregulate the production of PHA and glycogen production in order to increase fluxes towards other carbon storage compounds of interest [66], such as succinate [64]. However, the CRISPRi based gene editing to overexpress PHB biosynthetic genes has not been reported. While the CRISPR-based editing allows the creation of marker-less knockouts and knock-ins. Thus, in the future, the cyanobacterial strains produced might be considered commercially sustainable and safe for outdoor cultivations and CO_2_ sequestration. 

### 3.2. Process Design and Bioprocess Improvement Strategies

Nutrient deficiency or stress, mainly in terms of nitrogen or phosphorus limitation, stimulates the accumulation of PHB in cyanobacteria. Besides the strain engineering and improvement approach, various reports have discussed other factors, which can facilitate superior growth and productivity in cyanobacteria. Herein, the most important routes for improvement of PHB production in cyanobacteria are listed. 

#### 3.2.1. Media and Cultivation Conditions

Like all other bioprocesses, PHB production from cyanobacteria is mainly influenced by the cultivation parameters and nutrient supply. The importance of defined cultivation conditions used to obtain highly productive process for cyanobacteria and microalgae has been previously discussed [32,67,68]. Cyanobacterial growth requires a high concentration of essential nutrients, such as nitrogen, phosphorus, sulfur, potassium, magnesium, iron, and some traces of micromolecules. The supply of nutrients like nitrogen and phosphorus in limiting concentration is important for the production of PHB. Therefore, media optimization plays an important role to maximize the PHB productivity and lower production costs. Regarding optimized nitrogen concentration in the media, it was shown by Coelho et al. that 0.05 g L^−1^ nitrogen in the media results in the production of up to 30.7% (DCW) of PHB in *Spirulina* sp. LEB 18. Further optimization of nitrogen content to 0.22 g L^−1^ in the media increased the PHB content in spirulina sp. LEB 18 to 44.2% (DCW) [69]. However, the impact of nitrogen optimization on the volumetric or specific productivities were not reported in both cases. The optimization of media components, nitrogen, and phosphorus in the case of *Synechocystis* sp. PCC 6714 increased volumetric as well as specific production rates, both in the case of biomass growth and PHB content [62].

Besides media components, other key parameters influencing growth and PHB production in cyanobacteria are cultivation conditions, such as temperature, pH, light intensity, or light/dark cycles. Furthermore, production of the copolymers can be tailor-made by using co-substrates and varying the cultivation conditions, such as temperature and pH [70]. Various studies have used the statistical design of experiments (DoEs) in order to optimize the media as well as the cultivation conditions [31,32,71]. The DoEs are used to minimize the error in determining the influential parameters, allowing systematic and efficient variation of all factors [72]. Table 3 summarizes the cultivation parameters and the nutrient limitation used for cyanobacterial PHB synthesis. 

Two primary challenges of entering cyanobacterial PHB into the market are the concern of the sustainability of the production process and the high production costs of fresh water and nutrients. One solution could be to use waste streams like agricultural effluents with high nitrogen and phosphorus contents. Therefore, the production of the polymer is accompanied by the removal of nutrients from the water. On the other hand, the undefined substrate may raise new challenges that then need to be resolved [79]. Various reports have shown production of cyanobacterial PHB using waste streams. Troschl et al. have summarized the list of cyanobacterial strains cultivated on agro-industrial waste streams and anaerobic digestants to produce PHB [23]. One example is the cultivation of the diazotrophic cyanobacterial strain *Aulosira fertilisima* under a circulatory aquaculture system that resulted in increased dissolved oxygen levels during the cultivation period and the complete removal of nutrients, such as ammonia, nitrite, and phosphate, within 15 days of cultivation, yielding an average PHB content of 80–92 g m^−3^ [80]. This report, along with other previously shown studies [79,81,82,83,84], clearly shows the potential of cyanobacterial PHB production for wastewater treatment facilities. 

#### 3.2.2. PHB Production Using Mixed Photosynthetic Consortia

Another approach used for PHB production is the feast-famine strategy, which uses a mixed consortium of algae and cyanobacteria [85,86,87,88]. During this regime, the feast operation consists of a mixed culture of cyanobacterial consortium cultivated in a sequencing batch reactor (SBR) without aeration using acetate as a carbon source and light as an energy source [86]. During the famine phase, the NADH or the NADPH reserves of the cell is consumed using the oxygen produced by the algae cells present in the consortia leading to accumulation of around 20% (DCW) PHA [89]. Furthermore, maximum polymer content of 60% (DCW) of PHA was produced by a photosynthetic mixed culture in a permanent feast regime using high light intensity [86]. The anaerobic dark energy generation’s capability of cyanobacteria is already been known [90]. Some cyanobacteria have also been known for their fermentation capability at the expense of their carbohydrate reserves [91]. The axenic dark feast conditions facilitated the acetate uptake, increasing the productivity significantly (up to 60%) (DCW), as the famine phase was eliminated [85,86]. The anaerobic fermentation of cyanobacteria to produce PHB has a potential, while the need for sterilization and aeration is eliminated, reducing also the energy costs. However, the source and cost of the substrate used remains a cost driver issue. 

#### 3.2.3. PHB Production Using Mixed Feed Systems

Production of PHAs in cyanobacteria can occur during phototrophic growth, using CO_2_ as a sole carbon source and light energy, and also during heterotrophy, when using sugar supplementation. It has been estimated that the carbon substrate in a large-scale manufacturing context would constitute approximately 37% of the total production costs [27,92]. However, in order to cope with the low phototrophic PHB productivity in cyanobacteria, various studies have used supplementation of other carbon sources. The mini-review by Singh and Mallick has summarized the wild-type and recombinant cyanobacterial strains, their PHA content, and the substrate used for the biosynthesis of the biopolymer [24]. However, in most reported cases [34,46,93,94,95,96,97] of heterotrophic PHB production, the biomass concentrations produced are less than 1 g L^−1^ and increases in volumetric productivities are not described. Even though the PHB productivity increases in terms of biopolymer content (%DCW) using mixed feed systems, the use of external carbon substrates increases the production costs and also raises the question of the economic feasibility of PHB production from cyanobacteria. As long as heterotrophic organisms produce PHB at much higher rates than cyanobacteria, the only sense for commercialization of cyanobacterial PHB would be the sustainability. Therefore, research must focus on improving the phototrophic PHB production with the aim of increasing CO_2_ uptake rates of cyanobacteria, with the support of viable bioprocess technology tools. 

#### 3.2.4. CO_2_ Sequestration

CO_2_ is a major greenhouse gas; its emission into the atmosphere has gradually increased in the past decades, causing global warming and its associated problems [98]. Carbon contributes to all organic compounds and is the main constituent of cyanobacterial and all biomass, amounting to up to 65% of DCW [79]. The industrial production of PHB that uses CO_2_ feedstocks helps reduce the environmental impacts of CO_2_ emission. Various studies have shown that an increase in CO_2_ concentration during a cyanobacterial cultivation may increase the production of carbon reserve compounds, such as PHB. Markou et al. showed that an increase in carbon content leads to the production of carbon reserve compounds, such as lipids and PHAs [79]. The increase in the concentration of the carbon source also increased biopolymer accumulation in cyanobacterial strain *Spirulina* sp. LEB 18 [77]. However, what has not been discussed in the literature thus far are the effects of the day-night cycle on the CO_2_ uptake rate and the productivity of carbon reserve compounds in cyanobacteria. Since CO_2_ fixation occurs during the light phase, the total productivity and CO_2_ sequestration rate will be lower in outdoor cultivations. During the dark phase, CO_2_ utilization is minimized and the productivities are lowered and some carbon reserve molecules, such as glycogen, degrade. Other methods would need to be used to temporarily sequester CO_2_ as a carbonate species during nighttime, which could then be utilized by cyanobacteria when the light is available again [99]. 

## 4. Production Strategies

The economic efficiency of any production process is indicated by the productivity, which comprises of growth rates, specific production rates, and the biomass concentration of the culture. Therefore, the economic efficiency of the production process will increase only when the mentioned parameters are improved. Once the strain and cultivation parameters are selected and optimized for the production process, the process performance can be considered and analyzed.

### 4.1. Cultivation Modes

Cyanobacteria producing PHA have been classified into two groups based on the culture conditions required for efficient polymer synthesis: group one requires a limitation of an essential media component for PHA synthesis; the second has no requirement for nutrient limitation for the accumulation of the polymer [100]. For industrial production of PHB, the second group is favorable for growth as it is accompanied by polymer synthesis. 

In general, cultivation of cyanobacteria for the production of PHB can be done using various cultivation modes. The most common approach is using batch cultivation, in which the production of PHB is induced by a limiting nutrient or, in an ideal case, the production of the polymer becomes growth dependent. For the batch cultivation with the group one cyanobacteria, the concentration of nitrogen and phosphorus in the media play the key-role facilitating biomass growth, and their limitation trigger PHB synthesis. Thus, in such a process the cell growth is maintained without nutrient limitation, until the desired concentration is reached. Then, an essential limitation allows for efficient polymer accumulation. So far, a few studies have focused on optimizing the nutrients for the batch production of PHB in large-scale; others mainly have been done in flasks. Batch cultivation of *Synechocystis* sp. PCC 6803 using a nitrogen concentration of half of the optimal BG-11 media, showed 180 mg L^−1^ PHB from CO_2_ [74]. The maximum PHB content of 125 mg L^−1^ was obtained for the non-sterile batch cultivation of *Synechocystis* sp. CCALA192 in a 200-L tubular photobioreactor [42]. Moreover, PHB was produced using optimized media in a one-liter tank reactor for the wild-type cyanobacterial strain *Synechocystis* sp. PCC 6714 obtained 640 mg L^−1^ of polymer [62].

The other common strategy for the cyanobacterial PHB production is using the SBR mode of operation, where growth and production occur in different reactors. In the growth vessel, media components are provided in abundance to facilitate maximum biomass production. In the induction photobioreactor, one or more media components are limited facilitating PHB biosynthesis. During the induction, residual biomass concentration remains more or less constant, while cell concentration increases only by intracellular polymeric accumulation [101]. In order to facilitate higher productivities, both reactors can operate as chemostats. Thus far, no reports of cyanobacterial PHB production in SBR or chemostat mode have been described.

### 4.2. Cultivation Systems

Another challenge in commercial cyanobacterial production is associated with biomass production. There are three main production systems used for large-scale cultivation of microalgae and cyanobacteria. The most basic approach for the cultivation of photosynthetic organisms is the use of large natural locations, which is mostly done for microalgae such as *Dunaliella*. Releasing into natural locations is regarded as a deliberate release into the environment, since there are no effective protective measures to prevent the microalgae from entering the surroundings [60]. The other approach is the use of open raceway pond systems, which has been commonly applied worldwide. When these raceway ponds are used outdoors, the cultivation are regarded as a deliberate release, so the spread of genetically modified organisms cannot be excluded in this case [60]. Even though these systems are economically feasible, the maintenance of monocultures and improving productivity are the main bottlenecks associated with such cultivations. The use of an open pond system has so far been reported in a wastewater treatment facility, containing fish pond discharge that uses the cyanobacterial strain *Aulosira fertilissima*, which shows a PHB productivity of up to 92 g m^−3^ [80]. The third system is the sophisticated, closed production system: photobioreactors (PBR). These systems can be both placed in greenhouses to obtain more defined cultivation conditions or be installed outdoors. PBR systems are more flexible for the needs of the cultivation process and the desired species. The industrial-scale production of photoautotrophic cyanobacterial PHB has not been widely reported in photobioreactors. The various photobioreactor systems used to cultivate cyanobacteria is given by Koller et al. [102]. Yet Troschl et al. has described the cultivation of *Synechocystis salina* CCALA192 in a 200-L tubular photobioreactor for the production of PHB from CO_2_ [23]. The maximum PHB productivity obtained under nitrogen limitation was 6.6% (DCW), while the volumetric and specific productivities were not reported in this case. Moreover, a mixed consortium of wastewater born cyanobacteria was cultivated in a 30-L PBR, showing a maximum productivity of 104 mg L^−1^ under phosphorus limitation [103]. 

## 5. Process Monitoring and Control

Today the most commonly used method for accurate determination of PHAs in bacterial cultivations is gas chromatography (GC) [104] or high-performance liquid chromatography (HPLC) [105,106]. These methods involve hydrolysis, subsequent methanolysis, or propanolysis of the PHAs in whole cells, in the presence of sulfuric acid and chloroform [107]. These extraction methods are laborious, time-consuming, and the optimum time of harvest might be lost due to the time needed for the analysis. Other methods for PHA analysis include gravimetric, infrared spectroscopy of chemically extracted PHB, fluorimetry, and cell carbon analysis [107,108,109]. It is necessary to develop viable analytics to help the development of an efficient commercial production process that enables monitoring and control of production, along with a rapid feedback on the state of the process.

Fourier transform infrared (FTIR) spectroscopy has been applied to determine the chemical composition of cyanobacteria with major cellular analytes, such as proteins, lipids, polysaccharide, nucleic acids, and PHAs [107,110]. It has been shown that FTIR spectroscopy can monitor water-soluble extracellular analytes in fermentation systems, as well as being an indirect method to determine the stage of fermentation by monitoring the physiological state of the cells [111]. Various studies have shown the potential of FTIR spectroscopy for determination of intracellular PHA contents in various microorganisms [107,112]. In the same direction, Jarute et al. have introduced an automated approach for on-line monitoring of the intracellular PHB in a process with recombinant *E. coli*, which uses stopped-flow attenuated total reflection FTIR spectroscopy [113]. In the case of cyanobacteria, there exists no such studies reporting on-line or at-line determination of intracellular carbon compounds, such as PHAs and glycogen. The measurements used and the parameters controlled in microalgae processes are specific in-line probes, such as pO_2_, pCO_2_, pH, and temperature. In cyanobacterial industrial processes, spectroscopic measurement techniques, such as FTIR, can be used for monitoring, controlling the production, and determining the time of harvest. The on-line determination can also identify the limitation time and the limiting components based on the cell physiology, thus helping to make the cyanobacterial PHA production robust and manageable.

## 6. Production Scenarios

In order to compete with synthetic and other starch-based polymers in the market, the cost of cyanobacterial PHB needs to be reduced significantly. Yet, no economic analysis has been done to estimate the production costs of phototrophic PHB production. It has been reported that the cost of PHB production from heterotrophic organisms is in the range of 2–5 € kg^−1^ [114]. This value is still much higher than the estimated cost of petrochemical-based mass polymers like PE, PP, or PET, which is around 1.2 € kg^−1^ [114] and less. Taking into account the much lower time space yield and the biomass productivity in cyanobacteria and complications associated with the downstream processing, the cost associated with the production of PHB in cyanobacteria could be higher than that of heterotrophic microorganisms (>5 € kg^−1^). Typically, more than 4.3 kg of sugar is needed to produce 1 kg of PHB [85]. Nevertheless, higher yields of product per substrate consumed have also been reported, showing values of 3.1 kg sucrose/kg PHB and of 3.33 kg glucose/kg of the polymer [15,115]. In this context, the substrate costs can be avoided by photoautotrophically produced PHB by cyanobacteria. However, the lower productivities of cyanobacteria will still increase the costs significantly. Among the main factors contributing to the cost of PHA production are equipment-related costs, such as direct-fixed-capital-dependent items, overheads, and some labor-dependent factors, which considerably increase with a decrease in productivity [27,101]. Therefore, for the production of the same amount of PHA per year, the process with lower productivity requires larger equipment [27,116]. To that end, one approach could be to reduce the costs associated with the building of photobioreactors. This can be accomplished by simplifying the design and the material used for the production of photobioreactors and their energy consumption [22]. Another alternative to increase the size of the facility or reactor while also reducing production costs is to use open pond raceways and wastewater born cyanobacteria instead of fresh water strains. However, it should be taken into account that the increase in volume will directly increase the effort associated with the downstream processing [25]. 

Moreover, it has been shown that using industrial flue gases may reduce the production cost of cyanobacterial biomass to around 2.5 € kg^−1^, while using wastewater can decrease the costs further, to less than 2 € kg^−1^ [22,117]. Therefore, as already discussed, wastewater streams with high carbon, nitrogen, and phosphorus that are mix-fed with CO_2_ from industrial flue gases, can be used to make the PHB production from cyanobacteria more efficient. Furthermore, producing several chemicals from the same microalgae feedstock could potentially make the production of multiple commodity chemicals from a biological resource economically viable [92]. 

## 7. The Remaining Challenges in Photosynthetic PHB Production

Current industrial PHA production processes rely mostly on the availability of agricultural resources, which are unsustainable (compare the food versus fuel discussion with first-generation biofuels) and leave a large ecological footprint [65]. 

In the case of cyanobacterial, PHA production research has mainly focused on genetic engineering to increase productivity, which mainly reports as higher % DCW polymer content. The studies have rarely reported an increase in photosynthetic efficiency or an increase in the specific growth rates and production rates. So far, very few studies have shown the use of wastewater-open pond systems for the production of PHAs. For a recent review of PHA production, see Koller et al. [118].

## 8. Outlook

Currently, the global research efforts directed towards individual aspects of cyanobacterial PHA production mainly focus on improved strains and recovery processes. Although various challenges are associated with the efficiency of the cyanobacterial PHA productivity and the extraction and purification of PHAs, optimization of each step separately will waste considerable effort and result in overall sub-optimality [27]. With respect to commercialization and scale-up of the cyanobacterial PHA production, the view of the whole processes needs to be considered. More attention towards sustainable and viable upstream processing may help to reach an economic PHA production point. Cyanobacterial PHA production, from an economic point of view, will only make sense if a continuous process can be achieved, especially using waste streams as a carbon source and for the media. The process can then be coupled with the bioremediation of agricultural and industrial effluents. Thus far, some wild-type and improved cyanobacterial strains are reported with PHB content which is mostly cultivated under controlled, defined, and sterile lab conditions. For production in industrial scale that is done under unsterile conditions, only *Nostoc moscorum* as an example is reported. Other strains are not tested or can hardly tolerate the harsh outdoor conditions. Although we have emphasized the importance of optimized media and cultivation conditions on PHA productivity, sustainable and viable commercial processes conducted under unsterile conditions using waste streams and open systems are required. Research needs to focus on screening for more robust strains, such as wastewater born mixed-cultures that can tolerate fluctuations in cultivation conditions like pH, temperature, salinity, and media composition. Furthermore, the durable strains for which production of PHB is associated with biomass growth and therefore the time-spaced yield will be improved.

PHA shows both the advantages of biobased carbon content and full biodegradability. In addition, cyanobacterial PHA can be more sustainable and more cost effective in the marine environment and when compared to PHA from carbohydrate fermentation. It can be a carbon-negative material, making the process not only attractive for PHA converters and users, but also for CO_2_ emitters, like power stations. There are plenty of medium-size CO_2_ point sources, e.g., biogas production facilities, where the CO_2_ could be used in an adjacent cyanobacterial PHA factory erected on the non-arable land. Preferably, a biorefinery approach would be executed, where valuable compounds such as phytohormones and pigments are extracted from the cyanobacteria; then. PHA and biomass is anaerobically digested in a biogas plant, yielding a cost-effective and fully integrated process. 

It is expected that over the next two decades there will be a shift toward more recycling of fossil-based and conventional plastics, with an accompanying reduction in material variety to facilitate collection, processing, and reuse. Moreover, we will see a maturing of the bioplastics industry, with more applications being developed with bioplastics, other than “gimmick” giveaways and small household and kitchen tools. Due to the unique and interesting property set of PHA, it can be anticipated that these materials, particularly PHB and its copolymers such as PHBV, will gain significance.

## Figures and Tables

**Table 1:** Examples of wild-type cyanobacterial strains, with reported poly (3-hydroxybutyrate) (PHB) content, the carbon source, and growth conditions used for the production of the polymer.

Cyanobacteria	PHB Content (% DCW)	Substrate	Production Condition	Polymer Composition	Reference
*Synechocystis* sp. PCC 6803	38	Acetate	P limitation and gas exchange limitation ^1^	PHB	[31]
*Synechocystis* sp. PCC 6714	16	CO_2_	N ^2^ and P ^3^ limitation	PHB	[32]
*Spirulina* platensis	6.0	CO_2_	Not given	PHB	[33]
*Spirulina platensis* UMACC 161	10	acetate and CO_2_	N starvation	PHB	[34]
*Spirulina maxima*	7–9	CO_2_	N and P limitation	PHB	[35]
Gloeothece sp. PCC 6909	9.0	acetate	Not given	Not specified	[36]
*Nostoc moscorum Agardh*	60	acetate and valerate	N deficiency	PHB-co-PHV	[37]
Nostoc moscorum	22	CO_2_	P starvation	PHB	[38]
*Alusira fertilisima* CCC444	77	fructose and valerate	N deficiency	PHB-co-PHV	[39]
*Alusira fertilisima* CCC444	85	citrate and acetate	P deficiency	PHB	[40]
*Synechocystis* PCC 7942	3	CO_2_	N limitation	PHB	[41]
*Synechocystis* PCC 7942	25.6	acetate	N limitation	PHB	[41]
*Synechocystis* sp. CCALA192	12.5	CO_2_	N limitation	PHB	[42]
*Anabaena Cylindrica*	<0.005	CO_2_	Balanced growth	PHB	[43]
*Anabaena cylindrica*	2.0	propionate	N limitation	PHB + PHV	[43]
*Synechococcus elongatus*	17.2	CO_2_ and sucrose	N deficiency	Not specified	[44]
*Caltorix scytonemicola* TISTR 8095	25	CO_2_	N deficiency	PHB	[45]

^1^ gas exchange limitation = limitation of gas transfer to the culture vessel. ^2^ N = nitrogen. ^3^ P = phosphorus.

**Table 2:** Strategies to increase PHB biosynthesis yield.

Cyanobacterial Strain (Recombinant)	PHB Content (% DCW)	Genetic Tool Used	Production Conditions	References
*Synechococcus* sp. PCC 7942	1.0	Defective in glycogen synthesis	CO_2_	[53]
*Synechococcus* sp. PCC 7942	26	Introducing PHA biosynthetic genes from *C. necator*	Acetate and nitrogen limitation	[41]
*Synechocystis* sp. PCC 6803	26	Overexpression of native *pha* genes	CO_2_ and nitrogen deprivation	[46]
*Synechocystis* sp. PCC 6803	11	Introducing PHA biosynthetic genes from *C. necator*	Acetate and nitrogen limitation	[54]
*Synechocystis* sp. PCC 6803	14	Overexpression of PHA synthase	Direct photosynthesis	[55]
*Synechocystis* sp. PCC 6803	12	Increasing acetyl-CoA levels	CO_2_	[56]
*Synechococcus* sp. PCC 7002	4.5	Introduction of GABA Shunt	CO_2_	[57]
*Synechocystis* sp.	35	Optimization of acetoacetyl-CoA reductase binding site	CO_2_	[58]
*Synechocystis* sp. PCC 6803	7.0	Transconjugant cells harboring expression vectors carrying *pha* genes	CO_2_	[59]

**Table 3:** Reported cultivation parameters and media limitation used for photoautotrophic PHB production in wild-type cyanobacterial strains.

Cyanobacterial Strain	Limiting Component	Temperature °C	pH	Light Condition	PHB Content % (DCW)	Cultivation Time (Days)	Volume (L)	References
*Synechocystis* sp. PCC 6803	N and P starvation	28–32	7.5–8.5	dark/light cycle	11	10	0.05	[31]
*Synechocystis* sp. PCC6803	N starvation	30	n.p	light	4.1	7	n.p	[73]
*Synechocystis* sp. PCC6803	N limitation	28	n.p	18:6	8	30	0.8	[74]
*Synechocystis* sp. PCC 6714	N and P limitation	28	8.5	light	16.4	16	1	[32]
*Synechocystis salina* CCALA192	Optimized BG-11 media ^4^	n.p ^5^	8.5	light	6.6	21	200	[23]
*Phormidium* sp. *TISTR 8462*	N limitation	28	7.5	light	14.8	12	n.p	[75]
*Calothrix scytonemicola* TISTR 8095	N deprivation	28	7.5	light	25.4	12	n.p	[75]
*Nostoc muscorum*	Growth associated ^6^	25	8.5	14:10	8.6	21	0.05	[76]
*Nostoc muscorum*	P depletion	22	n.p	light	10.2	19	n.p	[38]
*Spirulina* sp. LEB 18	Defined media ^7^	30	n.p	12:12	30.7	15	1.8	[77]
*Aulosira fertilissima*	P limitation	28	8.5	14:10	10	4	0.05	[40]
*Anabaena* sp.	n.p	25	8	14:10	2.3	n.p	0.1	[78]

^4^ Optimized BG-11 media = the optimized BG-11 media contains 0.45 g L^−1^ NaNO_3_ and leads to a self-limitation of the culture. ^5^ n.p = not provided. ^6^ Growth associated = the production of PHA was associated with growth and no media limitation was given. ^7^ Defined media = concentration of nitrate, phosphate and sodium bicarbonate was optimized.
